# Trends and frontiers of RNA methylation in cancer over the past 10 years: a bibliometric and visual analysis

**DOI:** 10.3389/fgene.2024.1461386

**Published:** 2024-10-14

**Authors:** Bo-Na Liu, Xiao-Li Gao, Ying Piao

**Affiliations:** Department of Oncology, General Hospital of Northern Theater Command, Shenyang, China

**Keywords:** RNA methylation, cancer, bibliometric analysis, m6A, noncoding RNA

## Abstract

**Purpose:**

To highlight the trends and frontiers of RNA methylation in cancer over the past 10 years.

**Methods:**

Research publications on RNA methylation in cancer were retrieved from the Web of Science Core Collection database. VOSviewer, CiteSpace, and Bibliometrix were used to conduct bibliometric and visualization analysis of countries, institutions, authors, journals, and keywords relevant to this field.

**Results:**

From 2014 to 2023, research on RNA methylation in cancer has developed rapidly, with an overall increase in the number of publications and citations. China (4320 papers, 115056citations), Sun Yat Sen University (274 papers, 15698 citations), and Zhang, Wei (48 papers, 893 citations) are respectively the countries, institutions, and authors with the highest number of published papers and citations. Frontiers in Oncology (182 papers, 2524 citations) and Molecular Cancer (69 papers, 9224 citations) are the journals with the highest number of published papers and citations in this field, respectively. Co-occurrence analysis of keywords indicates that the research topics can be divided into five clusters: Cluster one: The Role of RNA Methylation in Tumor Heterogeneity, Therapeutic Response, and Prognosis; Cluster two: The Role of Noncoding RNA in RNA Methylation and Tumors; Cluster three: Potential Therapeutic Targets of RNA Methylation in Tumors; Cluster four: The role of RNA methylation in tumor progression and metastasis: A case study of hepatocellular carcinoma and gastric cancer; Cluster five: Regulation mechanisms of m6A methylation in leukemia cell differentiation and tumorigenesis.

**Conclusion:**

This is the first comprehensive study using bibliometrics to analyze the trends and frontiers of RNA methylation in cancer over the past 10 years, pointing out promising research directions for the future and providing valuable references for researchers in this field.

## 1 Introduction

RNA methylation, as an important form of epigenetic modification, has been shown to be involved in the pathogenesis of various cancers (Jiang et al., 2021). Compared to traditional DNA methylation, RNA methylation can directly affect multiple biological processes, such as RNA stability, splicing, transport, and translation, thereby regulating gene expression. During tumorigenesis and tumor progression, aberrant changes in RNA methylation have been confirmed to be closely related to tumor cell proliferation, migration, invasion, and drug resistance (Chong et al., 2021; Bae et al., 2021).

RNA methylation is a post-transcriptional regulation that mainly modulates RNA processing and decay (Frye et al., 2018). It refers to the transfer of active methyl groups to target chemicals under the catalysis of methyltransferases without altering the RNA sequence composition (Dai et al., 2021). The dynamics and biological outcomes of all methylation events result from the activity of a complex protein machinery composed of writers, erasers, and readers. Over 150 types of RNA modifications have been identified. They are widely distributed in messenger RNA (mRNA), transfer RNA (tRNA), ribosomal RNA (rRNA), small non-coding RNA (sncRNA), and long non-coding RNA (lncRNA). With the advancement of high-throughput sequencing technology and the continuous improvement of chemical labeling and antibody techniques for specifically recognizing RNA methylation sites, researchers have been able to reveal the complex mechanisms of RNA methylation in tumors on a broader scale. Particularly, RNA methylation modifications such as N6-methyladenosine (m6A), N1-methyladenosine (m1A), and 5-methylcytosine (m5C) have shown significant changes in various types of tumors. These methylation events play extensive roles in regulating cell fate, and their crosstalk with other post-transcriptional modification (PTM) events exert reversible control over various cellular behaviors (Lanouette et al., 2014).

The functions of RNA methylation, particularly those involving RNA methyltransferases, exhibit complex and multifaceted roles in tumor development. RNA methylation typically leads to decreased expression of downstream targets, with the effect strength depending on the specific characteristics of the target genes. For instance, the m6A methyltransferase METTL3 upregulates HBXIP expression by inhibiting the tumor suppressor gene let-7g through m6A methylation, forming a positive feedback loop (HBXIP/let-7g/METTL3/HBXIP) that promotes cancer cell proliferation in breast tumors (Cai et al., 2018). METTL3 is overexpressed in colorectal, gastric, and liver cancers, promoting cancer cell proliferation by inhibiting the tumor suppressor SOCS2 (Jiang et al., 2020; Xu et al., 2020; Chen et al., 2018). The m5C methyltransferase NSUN2 is overexpressed in various tumors, such as breast cancer and colorectal malignancies (Chellamuthu and Gray, 2020). NSUN2 protein is upregulated in 34% of breast cancers (Frye et al., 2010). Pan-cancer analysis shows that NSUN2 expression correlates positively with its DNA copy number and significantly affects patient clinical outcomes (Manning et al., 2020). In colorectal cancer, NSUN2 overexpression promotes cell migration by methylating precursor pri-miR-125b2, preventing its processing into miR-125b, thus silencing oncogenes including GAB2 (Yuan et al., 2014; Yang et al., 2015). On the other hand, RNA methylation also exhibits potential tumor suppressive effects. For example, METTL3 overexpression can significantly inhibit renal cell carcinoma proliferation and migration by interfering with epithelial-mesenchymal transition and the PI3K-AKT-mTOR pathway (Li X. et al., 2017). Additionally, different RNA methylation modifications may synergistically regulate tumor states. m5C methylation triggered by NSUN2 and m6A methylation catalyzed by METTL3/METTL14 synergistically upregulate the key tumor suppressor p21 in senescent cells under redox stress (Li Q. et al., 2017).

Bibliometric analysis of scientific research literature is a process that quantitatively analyzes scientific research publications using mathematical and statistical methods. It features quantification, systematicity, and visualization, revealing the development status, trends, academic frontiers, and hot topics in a specific field. It has broad application value in academic evaluation, discipline trends, and policy decision-making (Ninkov et al., 2022). Bibliometric analysis provides bibliometric relationships of authors, organizations, countries, and references in the relevant research field, involving in-depth studies of publications using techniques such as co-word analysis, collaboration network analysis, and clustering analysis (Liu et al., 2024). By analyzing databases and literature characteristics to assess the development trends of the target discipline/scientific field, summarizing the development of specific research topics, and revealing hotspots, emerging trends, and contributions (Feng et al., 2023; Peng et al., 2022), many bibliometric studies have been successfully applied in clinical medicine and biomedical fields. Currently, there is no quantitative and qualitative introduction of RNA methylation research in tumors. This study aims to reveal the objective performance and progress of RNA methylation in tumors from 1 January 2014, to 31 December 2023, through bibliometric analysis, providing better insights into this research trend and forecasting future development prospects.

## 2 Materials and methods

### 2.1 Data source

The Web of Science Core Collection (WoSCC) database was selected as the data source for bibliometric analysis due to its wide recognition in previous studies. We downloaded relevant publication data from 2014 to 2023 in “plain text” format.

### 2.2 Data collection

(1). Retrieval strategy included: The search terms were determined by the TS (“topic,” including title, abstract, author’s keywords, and keywords Plus) as TS = ((RNA methylation) AND TS = (tumor OR tumour OR cancer OR carcinoma OR sarcoma OR neoplasm OR malignant)).
(2). Publication type limited to “article” or “review”.
(3). Publication date ranging from 2014 to 2023.
(4). Publication language limited to English only.
(5). The following information was collected: publications, countries, authors, affiliated institutions, published journals, references, cited references, and keywords.

### 2.3 Bibliometric and visualization analysis

**FIGURE 1:**
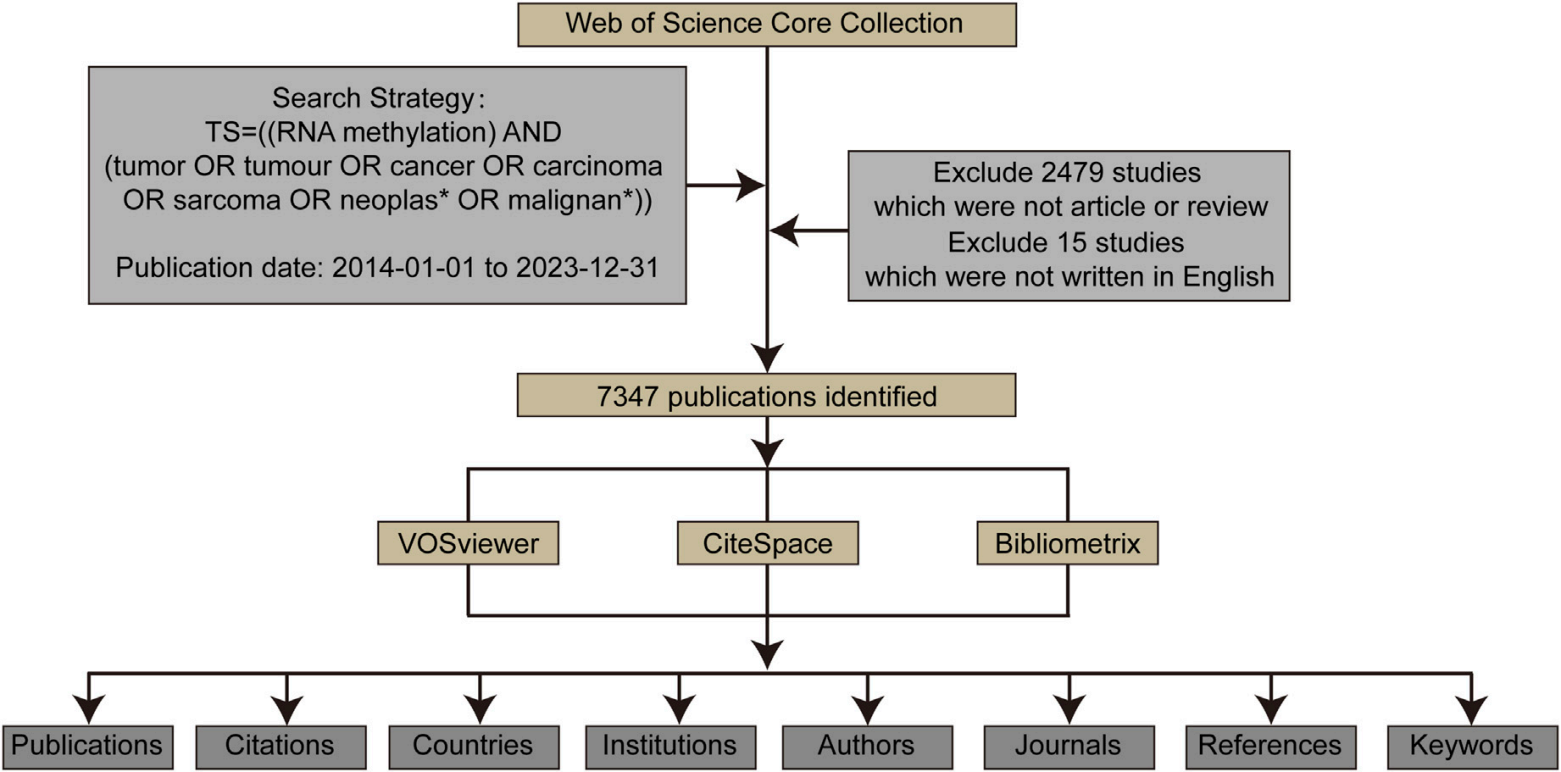
The data collection and retrieval strategy as well as the analytical process.

Bibliometric analysis and visualization of the publications retrieved from WoSCC were conducted using VOSviewer CiteSpace, and Bibliometrix (Figure 1). VOSviewer is a program used for constructing and viewing bibliometric maps based on similarity visualization technology (van Eck and Waltman, 2010). In this study, VOSviewer was used to analyze and visualize institutional, journal, and author collaborations related to RNA methylation, as well as keyword mapping in RNA methylation in cancer.

CiteSpace, a notable and influential tool for visualizing literary knowledge, was employed to intuitively map research hotspots and developmental trajectories, thereby forecasting the evolutionary patterns in various domains (Synnestvedt et al., 2005). Within this investigation, we harnessed the capabilities of CiteSpace to analyze and visually represent the research on RNA methylation in cancer, uncovering the foundational knowledge framework of this area and pinpointing the contemporary research tendencies.

Bibliometrix, a pioneering open-source tool crafted by Massimo Aria and Corrado Cuccurullo in 2017, was built utilizing the R language (Aria and Cuccurullo, 2017). While it offers co-citation analysis capabilities, Bibliometrix stands out for its advancements in data reduction techniques. In our study, we utilized the Bibliometrix R package to generate visualizations that highlight authors’ influence and production over time.

## 3 Results

### 3.1 Annual publication trends

**FIGURE 2:**
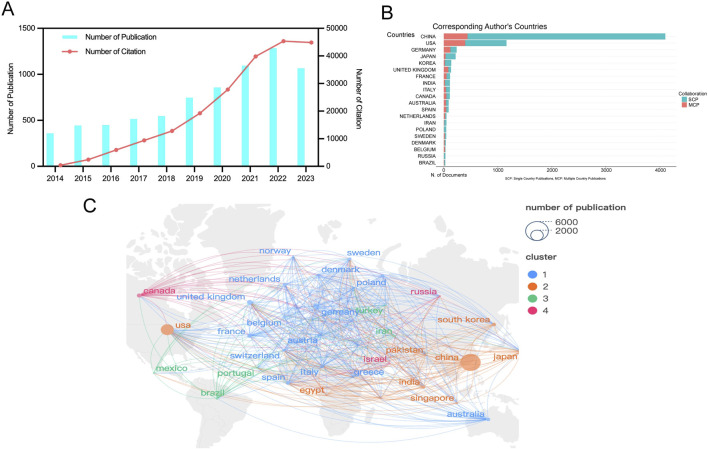
Geographic and temporal distribution of publications. **(A)** Annual publication and citation. **(B)** Top 20 countries with the largest number of publications. **(C)** Distribution and co-authorship network of countries/regions. Different nodes represent different countries, the connecting lines between nodes represent the cooperative relationship between countries, the size of the nodes represents the number of publications from that country, and the colors of the nodes and lines represent the different cooperation clusters formed among countries.

From 2014 to 2023, a total of 7,347 studies related to RNA methylation in tumors were retrieved from WoSCC. Figure 2A depicts the annual publication trends in this field. Over the past decade, there has been a steady increase in research related to RNA methylation in tumors. The annual global publication count rose from 358 in 2014 to 1,065 in 2023, an increase of 197.49%. Notably, there was an overall upward trend from 2014 to 2023, peaking in 2022 with 1,284 publications. Subsequently, the number of publications in 2023 was lower than the 2022 peak, at 1,065. Additionally, annual citation counts increased from 2014 to 2022, with a slight decline after 2023. This observation highlights significant attention and focus on this research area at an overall level. However, it is noteworthy that there appears to have been some challenges and difficulties in the field’s development in recent years.

### 3.2 Publication output and collaboration distribution of countries

RNA methylation in tumors is a globally recognized topic, with contributions from 93 countries. Table 1 outline the top ten countries by productivity. China (4,320 papers, 115,056 citations) emerged as the most productive country, followed by the USA (1,759 papers, 87,621 citations) and Germany (409 papers, 19,382 citations). Canada had the highest average citations per paper (61.13), followed by the USA (49.81) and Germany (47.39). Furthermore, we analyzed the distribution of corresponding authors of articles in this field across different countries, including both publications from a single country and publications resulting from multinational collaborations. The results indicate that China, the United States, and Germany rank among the top three countries in terms of the number of researchers engaged in the field of RNA methylation-related research in cancer (Figure 2B). This study used VOSviewer to analyze inter-country collaboration, revealing the international collaboration patterns in this field. A minimum publication threshold of 20 was set, and 35 countries met the criteria, forming a collaboration network with four clusters represented by different colors (Figure 2C). The USA ranked first in the number of collaborators (n = 65), followed by the United Kingdom (n = 53), Germany (n = 52), France (n = 49), and China (n = 46).

**TABLE 1:** The top 10 productive countries/regions.

Rank	Country	Publications	Citations	Average citations
1	China	4320	115068	26.64
2	United States of America	1759	87621	49.81
3	Germany	409	19382	47.39
4	United Kingdom	327	11553	35.33
5	Japan	303	9088	29.99
6	Canada	229	13999	61.13
7	France	202	8861	43.87
8	Italy	199	8653	43.48
9	South Korea	174	3892	22.37
10	Australia	171	7904	46.22

### 3.3 Publication output and collaboration distribution of institutions

**FIGURE 3:**
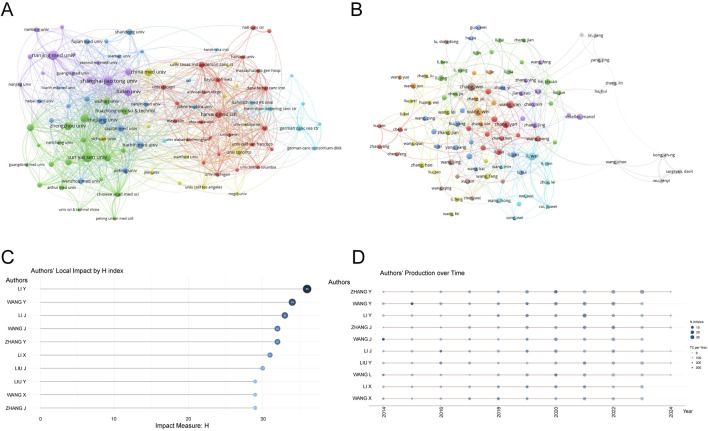
Analysis of institutions and authors. **(A)** VOSviewer visualization map of the co-authorship network of institutions. Different nodes represent different institutions, the connecting lines between nodes represent the cooperative relationship between institutions, the size of the nodes represents the number of publications from that institution, and the colors of the nodes and lines represent the different cooperation clusters formed among institutions. **(B)** VOSviewer visualization map of the co-authorship network of authors. Different nodes represent different authors, the connecting lines between nodes represent the collaborative relationship between authors, the size of the nodes represents the number of publications by that author, and the colors of the nodes and lines represent the different collaboration clusters formed among authors. **(C)** Authors’ local impact by H index. **(D)** Authors’ production over time.

A total of 6,288 institutions contributed to the research on RNA methylation in tumors. Table 2 introduces the top 20 institutions by productivity. Sun Yat-sen University (274 papers, 15,698 citations) became the most productive university, followed by Nanjing Medical University (249 papers, 9,977 citations) and Shanghai Jiao Tong University (244 papers, 7,926 citations). A minimum publication requirement of 30 was set, and 99 institutions met the criteria. VOSviewer was used to analyze collaboration among these institutions. The collaboration network, shown in Figure 3A, is composed of six clusters represented by different colors. The Chinese Academy of Sciences (n = 63), Sun Yat-sen University (n = 59), and Shanghai Jiao Tong University (n = 59) are at the center of the collaboration network with the most partners.

**TABLE 2:** The top 20 productive institutions.

Rank	Institution	Country	Publications	Citations	Average citations
1	Sun Yat Sen University	China	274	15698	57.29
2	Nanjing Medical University	China	249	9977	40.07
3	Shanghai Jiao Tong University	China	244	7926	32.48
4	Zhejiang University	China	188	11204	59.60
5	Fudan University	China	185	5086	27.49
6	Central South University	China	179	3187	17.80
7	China Medical University	China	171	7149	41.81
8	Zhengzhou University	China	160	3102	19.39
9	Chinese Academy of Sciences	China	142	7751	54.58
10	Huazhong University of Science and Technology	China	135	3057	22.64
11	Harbin Medical University	China	131	3666	27.98
12	Southern Medical University	China	131	2881	21.99
13	Tongji University	China	125	2941	23.53
14	Wuhan University	China	123	4791	38.95
15	Harvard Medical School	United States of America	113	8612	76.21
16	Capital Medical University	China	110	2891	26.28
17	Shandong University	China	110	2552	23.20
18	University of Texas MD Anderson Cancer Center	United States of America	109	8727	80.06
19	German Cancer Research Center	Germany	107	4367	40.81
20	Guangzhou Medical University	China	101	3229	31.97

### 3.4 Publication output and collaboration distribution of authors

From 2014 to 2023, a total of 44,387 authors contributed to the research on RNA methylation in tumors. Table 3 introduces the top 20 most productive authors. Zhang Wei (48 papers, 839 citations) is the most productive author, followed by Wang Wei (47 papers, 867 citations) and Li Wei (44 papers, 2,309 citations). VOSviewer was used for co-authorship analysis in the current research field. With a minimum publication requirement of 13, 102 authors met the criteria. Among them, 100 authors formed a network of eight clusters (Figure 3B) represented by different colors. Li Wei (n = 21), Zhang Yan (n = 19), and Wang Wei (n = 17) had the most collaborators and are at the center of the collaboration network. In addition, using bibliometrix, Figure 3C displays the top 10 authors with the highest H-index, partially representing their influence, while Figure 3D reflects that authors with high output have maintained a stable and continuous output in this research field over the past decade.

**TABLE 3:** The top 20 productive authors.

Rank	Author	Publications	Citations	Average citations
1	zhang, wei	48	839	17.48
2	wang, wei	47	867	18.45
3	li, wei	44	2309	52.48
4	zhang, yan	37	1109	29.97
5	wang, yan	37	736	19.89
6	esteller, manel	27	1541	57.07
7	wang, jing	26	1505	57.88
8	zhang, jing	26	705	27.12
9	zhang, hao	26	346	13.31
10	he, chuan	25	3344	133.76
11	li, xia	25	1223	48.92
12	li, xin	25	854	34.16
13	wang, yu	25	802	32.08
14	li, yan	25	553	22.12
15	wang, fang	24	1366	56.92
16	liu, yang	24	834	34.75
17	li, jing	24	723	30.13
18	wang, peng	23	1031	44.83
19	wang, lei	23	993	43.17
20	wang, xin	23	501	21.78

### 3.5 Source journal distribution

**FIGURE 4:**
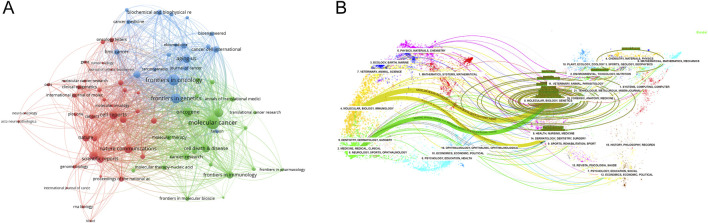
Analysis of source journals. **(A)** Citation analysis of source journals. The size of the nodes represents the citation intensity with other journals, while the connecting lines represent the citation relationships between journals. **(B)** Dual map overlay of journals. A dual-map overlay of journals shows the distribution of relationships between journals, citing journals on the left and cited journals on the right, with colored paths between them indicating citation relationships and their thickness indicating co-citation strength. The color corresponds to the time when the node was first co-cited.

VOSviewer was used to analyze the source journals of the publications. Table 4 lists the top 20 journals with the most related articles, with 62 or more publications. The journal with the most publications was Frontiers in Oncology (182 papers, 2,524 citations); the most cited journal was Molecular Cancer (69 papers, 9,224 citations). Among the top ten journals by publication count, the highest impact factors were Molecular Cancer (IF 37.3), Nature Communications (IF 16.6), and Nucleic Acids Research (IF 14.9). To provide a description of the citation patterns between relevant journals, we utilized VOSviewer to enhance the citation analysis among them. As shown in Figure 4A, the size of the nodes in the figure represents the citation intensity with other journals, while the connecting lines represent the citation relationships between journals. The results indicate that, in the field of methylation research in cancer, Molecular Cancer has the strongest citation relationship with other journals. The articles citing this journal primarily originate from Frontiers in Oncology, Frontiers in Cell and Developmental Biology, and Frontiers in Genetics, among others. In turn, the articles in Molecular Cancer primarily cite journals such as Oncogene, Seminars in Cancer Biology, and Molecular Therapy.

**TABLE 4:** The top 20 productive journals.

Rank	Journal	Publications	Citations	If 2022	JCR quartile 2022
1	Frontiers in Oncology	182	2524	4.7	Q2
2	Scientific Reports	169	3530	4.6	Q2
3	Frontiers in Genetics	150	1414	3.7	Q2
4	PLOS ONE	115	2475	3.7	Q2
5	Cancers	108	991	5.2	Q2
6	Nucleic Acids Research	99	7422	14.9	Q1
7	Cell Death & Disease	98	3740	9	Q1
8	Frontiers in Cell and Developmental Biology	93	1249	5.5	Q1/Q2
9	Nature Communications	92	6011	16.6	Q1
10	Oncology Letters	83	1019	2.9	Q3
11	Aging (US)	82	1373	5.2	Q2/Q2
12	Clinical Epigenetics	80	1864	5.7	Q1/Q2
13	Oncogene	79	4436	8	Q1/Q1/Q1/Q1
14	International Journal of Molecular Sciences	77	754	5.6	Q1/Q2
15	Frontiers in Immunology	73	989	7.3	Q1
16	Gene	72	916	3.5	Q2
17	Molecular Cancer	69	9224	37.3	Q1/Q1
18	BMC Cancer	65	1099	3.8	Q2
19	Oncology Reports	65	1411	4.2	Q2
20	Proceedings of the National Academy of Sciences of the United States of America	62	3547	11.1	Q1

Utilizing CiteSpace, another analysis was conducted to explore the citation patterns among journals publishing pertinent studies, resulting in a dual-map overlay visualization (Figure 4B). The left side of the figure portrays the citing journals, while the right side represents the cited journals. The curved line traversing from left to right signifies a citation-path connecting line, which highlights the flow and interconnection of knowledge across diverse research domains. As evident in Figure 4B, research on RNA methylation in cancer is primarily concentrated in disciplines like Molecular, Biology, Immunology, Medicine, Medical, and Clinical, and the literature citing this research also originates primarily from these same areas.

### 3.6 Most cited and Co-cited publications

Using Vosviewer, we analyzed the citation and co-citation patterns of publications within this research field. The results indicate that among the 7347 publications included in our study, 50 articles have been cited more than 380 times. Table 5 lists the top 10 most cited articles, with the Article titled “Mammalian WTAP is a regulatory subunit of the RNA N6-methyladenosine methyltransferase” by Xiao-Li Ping et al., published in Cell Research in 2014, receiving the highest number of citations (n = 1563).

**TABLE 5:** The Top 10 highly cited publications.

Rank	Title	First author	Source journal	Type	Publication year	Total citations	Ref
1	Mammalian WTAP is a regulatory subunit of the RNA N6-methyladenosine methyltransferase	Xiao-Li Ping	Cell Research	Article	2014	1563	Ping et al. (2014)
2	Recognition of RNA N6-methyladenosine by IGF2BP proteins enhances mRNA stability and translation	Huilin Huang	Nature Cell Biology	Article	2018	1548	Huang et al. (2018)
3	Inhibiting DNA Methylation Causes an Interferon Response in Cancer via dsRNA Including Endogenous Retroviruses	Katherine B Chiappinelli	Cell Research	Article	2015	1114	Chiappinelli et al. (2015)
4	The m (Lanouette et al., 2014)A Methyltransferase METTL3 Promotes Translation in Human Cancer Cells	Shuibin Lin	Molecular Cell	Article	2016	1077	Lin et al. (2016)
5	DNA-Demethylating Agents Target Colorectal Cancer Cells by Inducing Viral Mimicry by Endogenous Transcripts	David Roulois	Cell	Article	2015	910	Roulois et al. (2015)
6	RNA N6-methyladenosine methyltransferase-like 3 promotes liver cancer progression through YTHDF2-dependent posttranscriptional silencing of SOCS2	Mengnuo Chen	Hepatology	Article	2018	311	Chen et al. (2018)
7	m6A RNA Methylation Regulates the Self-Renewal and Tumorigenesis of Glioblastoma Stem Cells	Qi Cui	Cell Reports	Article	2017	897	Cui et al. (2017)
8	Perturbation of m6A writers reveals two distinct classes of mRNA methylation at internal and 5′sites	Schraga Schwartz	Cell Reports	Article	2014	895	Schwartz et al. (2014)
9	The N6-methyladenosine (m6A)-forming enzyme METTL3 controls myeloid differentiation of normal hematopoietic and leukemia cells	Ly P Vu	Nature Medicine	Article	2017	841	Vu et al. (2017)
10	FTO Plays an Oncogenic Role in Acute Myeloid Leukemia as a N6-Methyladenosine RNA Demethylase	Zejuan Li	Cancer Cell	Article	2017	762	Li et al. (2017c)

Additionally, the co-citation analysis revealed that the 213,970 co-cited documents among the studied publications include 121 references cited more than 100 times. Table 6 presents the top 10 most co-cited references, with the Article titled “N6-methyladenosine-dependent regulation of messenger RNA stability” by Xiao Wang et al., published in Nature in 2013, being the most co-cited reference (n = 462).

**TABLE 6:** The Top 10 highly co-cited publications.

Rank	Title	First author	Source journal	Type	Publication year	Total citations	Ref
1	N6-methyladenosine-dependent regulation of messenger RNA stability	Xiao Wang	Nature	Article	2013	462	Wang et al. (2014)
2	Topology of the human and mouse m6A RNA methylomes revealed by m6A-seq	Dan Dominissini	Nature	Article	2012	441	Dominissini et al. (2012)
3	N6-methyladenosine in nuclear RNA is a major substrate of the obesity-associated FTO	Guifang Jia	Nature Chemical Biology	Article	2011	363	Jia et al. (2011)
4	Comprehensive analysis of mRNA methylation reveals enrichment in 3′UTRs and near stop codons	Kate D Meyer	Cell	Article	2012	357	Meyer et al. (2012)
5	N (6)-methyladenosine Modulates Messenger RNA Translation Efficiency	Xiao Wang	Cell	Article	2015	350	Wang et al. (2015)
6	A METTL3-METTL14 complex mediates mammalian nuclear RNA N6-adenosine methylation	Jianzhao Liu	Nature Chemical Biology	Article	2013	331	Liu et al. (2014)
7	ALKBH5 is a mammalian RNA demethylase that impacts RNA metabolism and mouse fertility	Guanqun Zheng	Molecular Cell	Article	2013	325	Zheng et al. (2013)
8	RNA N6-methyladenosine methyltransferase-like 3 promotes liver cancer progression through YTHDF2-dependent posttranscriptional silencing of SOCS2	Mengnuo Chen	Hepatology	Article	2018	311	Chen et al. (2018)
9	The m (Lanouette et al., 2014)A Methyltransferase METTL3 Promotes Translation in Human Cancer Cells	Shuibin Lin	Molecular Cell	Article	2016	296	Lin et al. (2016)
10	Dynamic RNA Modifications in Gene Expression Regulation	Ian A Roundtree	Cell	Review	2017	288	Roundtree et al. (2017)

It is noteworthy that two articles have appeared in both the top 10 lists for citations and co-citations. These are the Article “RNA N6-methyladenosine methyltransferase-like 3 promotes liver cancer progression through YTHDF2-dependent posttranscriptional silencing of SOCS2” by Shuibin Lin et al., published in Molecular Cell in 2016, and the Article “The m (Lanouette et al., 2014) A Methyltransferase METTL3 Promotes Translation in Human Cancer Cells” by Mengnuo Chen et al., published in Hepatology. This underscores the significant contributions of these two articles to the field.

### 3.7 Keyword Co-occurrence analysis

**FIGURE 5:**
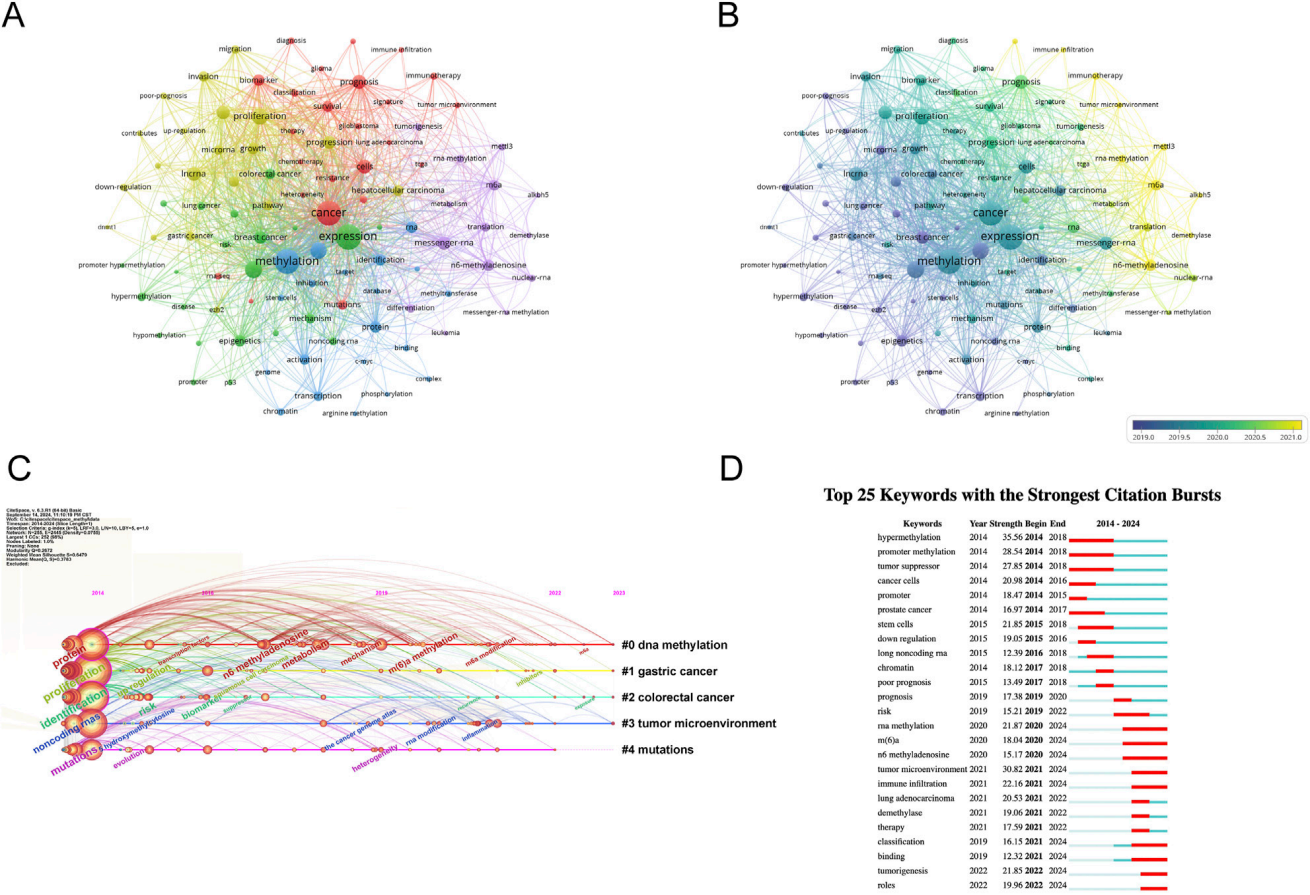
Analysis of dominant keywords. **(A)** The co-occurrence cluster analysis of the top 100 keywords. Each node represents a keyword, the connecting lines between nodes represent the co-occurrence relationship between two keywords, the size of the nodes represents the frequency of occurrence of the keyword, and the colors of the nodes and lines represent the different clusters formed by the keywords. **(B)** The overlay map of the top 100 keywords. The colors of the nodes and lines represent the average appearance year of the keywords. Warmer colors indicate a later occurrence, while cooler colors indicate an earlier occurrence of the keywords. **(C)** Timeline view for co-words analysis, The position of the nodes corresponds to the time of publication, the color of the ring represents the time of the citation, the thickness of the ring depends on the frequency of citations, and the lines between nodes represent co-citations. **(D)** Detection of top 25 keywords for the citation burst. Red bars mean that keywords are cited frequently in a certain period.

Keywords encapsulate the themes of publications, making high-frequency keywords suitable for co-occurrence analysis. In this study, VOSviewer was used to extract and cluster keywords. When the minimum occurrence frequency was set to 80, 100 keywords met the criteria. Table 7 shows the top 100 keywords by rank, frequency, and cluster affiliation. Figure 5A displays a network diagram of the top 100 keywords and their co-occurrence. Based on similarity, VOSviewer automatically grouped the keywords into five clusters, represented by different colors: red (Cluster 1), green (Cluster 2), blue (Cluster 3), yellow (Cluster 4), and purple (Cluster 5).

**TABLE 7:** Clusters of the top 100 Keywords.

Rank	Keywords	Cluster	Counts	Rank	Keywords	Cluster	Counts
1	expression	2	2253	51	tumorigenesis	5	182
2	methylation	3	2204	52	binding	3	181
3	cancer	1	2171	53	risk	2	179
4	gene	2	1057	54	glioblastoma	1	170
5	proliferation	4	946	55	promoter methylation	2	170
6	gene expression	3	944	56	rna-seq	1	167
7	prognosis	1	711	57	poor-prognosis	4	163
8	lncrna	4	704	58	methyltransferase	3	161
9	cells	1	671	59	diagnosis	1	159
10	messenger-rna	5	665	60	stem-cells	3	154
11	metastasis	4	632	61	ezh2	4	153
12	progression	4	605	62	epithelial-mesenchymal transition	4	152
13	breast cancer	2	582	63	overexpression	4	152
14	n6-methyladenosine	5	500	64	tumor microenvironment	1	148
15	identification	3	497	65	therapy	1	145
16	protein	3	474	66	association	2	142
17	epigenetics	2	463	67	metabolism	5	136
18	biomarker	1	462	68	promoter	2	131
19	rna	3	458	69	signature	1	125
20	growth	4	457	70	disease	2	124
21	m6a	5	445	71	inflammation	2	124
22	survival	1	421	72	target	3	124
23	hepatocellular carcinoma	4	418	73	complex	3	122
24	invasion	4	404	74	genome	3	122
25	colorectal cancer	2	391	75	glioma	1	121
26	activation	3	371	76	lung adenocarcinoma	1	121
27	apoptosis	4	370	77	p53	2	121
28	transcription	3	358	78	epigenetic regulation	2	118
29	pathway	4	335	79	5-methylcytosine	3	113
30	mechanism	2	333	80	database	3	113
31	microrna	4	326	81	demethylase	5	111
32	mutations	1	326	82	upregulation	4	110
33	gastric cancer	4	303	83	chemotherapy	1	108
34	translation	5	301	84	immune infiltration	1	108
35	differentiation	5	278	85	bioinformatics	1	104
36	migration	4	258	86	contributes	4	103
37	hypermethylation	2	254	87	hypomethylation	2	103
38	prostate cancer	2	241	88	phosphorylation	3	102
39	noncoding rna	2	240	89	receptor	2	101
40	lung cancer	2	239	90	leukemia	5	100
41	rna methylation	5	228	91	messenger-rna methylation	5	95
42	nuclear-rna	5	208	92	alkbh5	5	89
43	classification	1	207	93	tcga	1	87
44	landscape	1	199	94	c-myc	3	86
45	resistance	1	198	95	heterogeneity	1	84
46	mettl3	5	197	96	arginine methylation	3	83
47	inhibition	3	196	97	evolution	1	83
48	immunotherapy	1	195	98	promoter hypermethylation	2	82
49	chromatin	3	188	99	dnmt1	4	80
50	downregulation	4	184	100	inactivation	2	80

Cluster 1 comprises 24 keywords, with the top three most frequently occurring keywords being “cancer” (n = 2171), “prognosis” (n = 711), and “cells” (n = 671). Cluster 2 encompasses 22 keywords, where the top three keywords by frequency are “expression” (n = 2253), “gene” (n = 1057), and “breast cancer” (n = 582). Cluster 3 includes 20 keywords, featuring “methylation” (n = 2204), “gene expression” (n = 944), and “identification” (n = 497) as the three most cited keywords. Cluster 4 has 20 keywords, with “proliferation” (n = 946), “lncrna” (n = 704), and “metastasis” (n = 632) ranking among the top three in terms of occurrence. Lastly, Cluster 5 consists of 14 keywords, where “messenger-rna” (n = 665), “n6-methyladenosine” (n = 500), and “m6a” (n = 445) are the three most prominent keywords based on their frequency of mention.

To explore the temporal evolution of research hotspots, VOSviewer was used to analyze the average appearance year (AAY) of the top 100 keywords. Figure 5B shows the visualization of development based on their year of distribution, with the color in the circle adjusting to the year of publication. Yellow represents the 2021 interval, and dark blue represents the 2019 interval, indicating the time-based distribution of keyword usage. Yellow describes the types of keywords currently frequently used. We found that earlier keywords include “downregulation” (AAY: 2018.45), “apoptosis” (AAY: 2019.20), “progression” (AAY: 2020.1), and “mettl3” (AAY: 2021.2). Recently emerging high-frequency keywords include “immune infiltration” (AAY: 2022.01). The research focus has shifted from early studies on methylation regulation phenomena in chromosome development in breast and prostate cancer and their association with tumor disease progression, affecting biological behaviors such as tumor cell transcription, apoptosis, and invasion. As time has progressed, the current research direction is increasingly focused on linking tumor cells with immunotherapy to achieve clinical treatment translation.

### 3.8 Timeline view and keyword burst

The timeline view of keywords offers a glimpse into the evolving trends of research hotspots within a particular field over time. Leveraging the power of CiteSpace, we delved into the timeline analysis of RNA methylation research in cancer spanning the period from 2014 to 2023 (depicted in Figure 5C). The keywords were categorized into five distinct clusters, with the number of keywords in each cluster serving as a proxy for the significance of that particular topic within the research landscape. The ranking of these clusters is based on the number of citations they have garnered, reflecting their influence and relevance. Notably, the first four clusters cover a substantial portion of the timeline, suggesting that they have remained consistently prominent topics within the field.

The analysis of keyword bursts serves as a metric to gauge the level of acceptance and dissemination of pivotal research topics. Utilizing CiteSpace, we conducted a burst analysis on the key terms associated with RNA methylation research in cancer between 2014 and 2023. The outcome is depicted in Figure 5D, highlighting the top 25 keywords that exhibited the greatest burst intensity during this period. Keywords with the strongest burst intensity include “hypermethylation” (Strength = 35.56), “tumor microenvironment” (Strength = 30.82), and “promoter methylation” (Strength = 28.54), indicating the widespread acceptance of these topics. Recent keywords in a burst state include “roles” (2022–2024), “tumorigenesis” (2022–2024), “binding” (2021–2024), and “classification” (2021–2024), etc., indicating the current hotspots in these areas.

## 4 Discussion

This study employs bibliometric methods to delve into the key features of RNA methylation research in tumors by statistically analyzing the intrinsic connections between published works. This includes identifying influential countries, journals, research institutions, and authors. Our goal is to assess the academic contributions within this field, identify core literature, major research focuses, and current trends. In the discussion section, we will deeply analyze and discuss the main findings to provide valuable references and insights for researchers in the field of RNA methylation in tumors.

### 4.1 Trends and current status of global publications

Over the past decade, research on RNA methylation in tumors has experienced an accelerated development phase, as evidenced by the increasing number of related publications. Globally, 7,347 papers on RNA methylation in tumors have been published across 93 countries. China (4,320 papers) leads in this research area and plays a significant role in international collaborations, followed by the United States (1,759 papers) and Germany (409 papers). However, the average citation rate of Chinese papers (26.64 citations per paper) is lower than that of the United States (49.81 citations per paper) and Germany (47.39 citations per paper). Among the top 20 institutions, 85% are located in China, followed by the United States (n = 2, 10%) and Germany (n = 1, 5%). This phenomenon may be attributed to the leading scientific and economic strengths of these countries. In RNA methylation research in tumors, China demonstrates outstanding capabilities, which are not merely due to its strong economic background but also significant investments in the healthcare sector. National economic support and international collaborations will continue to promote in-depth exploration and comprehensive progress in this field.

In recent years, Sun Yat-sen University (274 papers, 15,698 citations) and Nanjing Medical University (249 papers, 9,977 citations) have led in academic paper publications in this field in China. However, when ranked by citations, 9 of the top 20 institutions are from China, 9 from the United States, and the remaining 2 from Canada and Germany. Furthermore, among the top 20 institutions with the highest publication output in this field, the University of Texas MD Anderson Cancer Center (with an average citation count of 80.06), Zhejiang University (59.60), and Sun Yat-sen University (57.29) exhibit the highest average citation rates, underscoring the significant scholarly impact and widespread attention garnered by their publications.

In terms of authors, Zhang Wei from China (48 papers, 839 citations) has published the most articles in the field of RNA methylation in tumors, while Wang Jing, also from China (26 papers, 1,505 citations), is the most cited author. Additionally, among the top 20 authors with the highest publication output, the authors with the highest average citation counts are He Chuan (133.76), Wang Jing (57.88), and Esteller Manel (57.07). This underscores the extensive attention and broad scholarly impact of their published works, thereby confirming their prominent positions within the field of RNA methylation research in cancer. Our research finds that the global investigation into RNA methylation in tumors has garnered widespread attention, with China and the United States being leaders in this field.

Additionally, this study constructed a collaboration network among countries, institutions, and authors to evaluate cooperation in this research area. The results show that the United States (n = 65), the United Kingdom (n = 53), and Germany (n = 52) have the most collaborative partners, indicating a preference for international partnerships among authors from these countries. The Chinese Academy of Sciences (n = 63), Sun Yat-sen University (n = 59), and Shanghai Jiao Tong University (n = 59) are central in the institutional collaboration network, having the most cooperative partners. This suggests that compared to other countries, China tends to prefer domestic institutional collaborations.

This study reviewed the main academic journals publishing research outcomes on RNA methylation in tumors. We recommend that researchers dedicated to this field consider these journals as primary platforms to showcase their academic achievements. Impact Factor (IF) (Zimmerman et al., 2022) and Journal Citation Reports (JCR) (Atallah et al., 2020) are powerful indicators of a journal’s influence. According to JCR ratings, 19 of the top 20 journals by publication volume in this field are in the Q1/Q2 quartiles, with 10 publishers from the United States, 5 from Switzerland, and 4 from the United Kingdom. Among them, Molecular Cancer significantly leads with an IF of 37.3 and a total of 9,224 citations, underscoring its importance and academic value in the field of RNA methylation and tumor research. Despite China’s significant contributions to this research area, no Chinese publisher ranks among the top 20 journals.

### 4.2 Research hotspots in RNA methylation in tumors

Co-occurrence analysis is a valuable tool for identifying specific research topics and trends within a particular research field. Keywords represent the core content of the research, while keyword frequency reflects the influence of keywords in a specific field. We conducted co-occurrence clustering analysis on the top 100 keywords and identified five research clusters in RNA methylation in tumors from 2014 to 2023. Each cluster corresponds to a specific research theme.

#### 4.2.1 Cluster one: the role of RNA methylation in tumor heterogeneity, therapeutic response, and prognosis

This cluster includes 24 keywords, such as cancer, prognosis, cells, biomarker, survival, mutations, classification, landscape, resistance, immunotherapy, glioblastoma, and diagnosis. The central theme of this cluster can be summarized as the role of RNA methylation in cancer and its impact on prognosis, diagnosis, and treatment (with glioblastoma as a representative).

To date, numerous studies have confirmed the close association of various RNA methylation modifications with the occurrence, development, and prognosis of tumors. The internal modifications of mRNA include N6-methyladenosine (m6A), 5-methylcytosine, N1-methyladenosine, and internal 7-methylguanosine (m7G) (Frye et al., 2018). The most typical RNA modification is methylation at the 6th position of adenine (m6A). It involves various aspects of RNA metabolism, such as stability, translation, splicing, transport, and localization, all of which have been found to affect various aspects of tumors (Xu Y. et al., 2023). m6A methylation accounts for over 80% of RNA methylation types (Chen et al., 2019; Deng et al., 2018). According to large-scale glioblastoma genomics and clinical data from the Chinese Glioma Genome Atlas (CGGA) and The Cancer Genome Atlas (TCGA), the main regulatory factor of m6A methylation, METTL3, shows differential expression in glioblastomas and is closely associated with disease progression (Chai et al., 2019). Knocking down METTL3 in mouse models can inhibit tumor growth. The level of m6A methylation is significantly reduced in glioblastoma tissues, while upregulation of m6A methylation in glioblastoma stem cells (GSCs) significantly reduces cell proliferation and migration by regulating the level of HSP90 (Li et al., 2019a). In neurosphere assays of human glioblastoma cell lines, silencing METTL3 reduces the expression of glioblastoma reprogramming factors POU3F2, SOX2, SALL2, and OLIG2, and inhibits glioblastoma cell proliferation (Visvanathan et al., 2018; Chang et al., 2021a). In METTL3-silenced GSCs, transcripts containing METTL3-dependent m6A peaks are upregulated at the transcriptional level, indicating that m6A modification mediates the stability of these transcripts. Gene Set Enrichment Analysis (GSEA) shows a significant decrease in genes associated with oncogenic pathways after METTL3 silencing, including MYC, mTORC1, E2F, TGF-β/NF-κB, as well as cell cycle and DNA repair pathways. This supports the view that METTL3-mediated m6A modification is necessary for maintaining the high expression of genes required for GSCs and tumorigenesis (Li et al., 2019b). Additionally, RNA sequencing found that genes enriched in m6A-regulated genomes in METTL3-silenced GBM cell lines are related to cancer pathways, including the apoptosis signaling pathway (Visvanathan et al., 2018; Shi et al., 2021).

Tumor heterogeneity refers to the diversity within tumors at the genetic, epigenetic, and phenotypic levels, which helps tumors adapt to environmental conditions and evade treatment (Fabbri et al., 2021). Single-cell m6A sequencing shows highly heterogeneous m6A modifications between single cells and throughout the cell cycle (Tegowski et al., 2022). m6A modification, by regulating the methylation status of nuclear RNA and mRNA, leads to high heterogeneity in tumor cells and affects the maintenance and differentiation of glioblastoma stem cells (GSCs), thereby increasing the diversity within tumors. Studies have shown that downregulation of methyltransferases METTL3 or METTL14 significantly promotes the proliferation, self-renewal, growth, and tumorigenesis of GSCs (Pan et al., 2018). Conversely, overexpression of METTL3 inhibits the proliferation and self-renewal of GSCs. It has been explicitly pointed out that m6A modification may be a mechanism by which GSCs adapt to environmental conditions and develop therapy resistance (Li et al., 2022; Xu Z. et al., 2023). In temozolomide-resistant glioblastoma stem-like cells, m6A modification significantly increases, and temozolomide treatment induces upregulation of METTL3, leading to increased m6A methylation (Li et al., 2021).

The role of m6A modification in tumor treatment response is of great concern. Changes in the activity of key methylases such as METTL3 and ALKBH5 can significantly affect the response of tumor cells to chemotherapy and radiotherapy. For example, overexpression of METTL3 may enhance the sensitivity of tumor cells to certain chemotherapy drugs, while high expression of ALKBH5 may lead to resistance. Additionally, m6A modification affects the response of tumor cells to immunotherapy by regulating the expression of immune checkpoint genes. By regulating m6A modification, it is hoped to improve the efficacy of tumor treatment and overcome resistance issues. It is worth noting that GSCs with silenced METTL3 are more sensitive to radiotherapy (Deacon et al., 2023). These findings suggest the enormous potential of m6A modification and its regulatory enzymes in tumor treatment.

The m6A modification is closely related to the prognosis of cancer patients. Studies have shown that the level of m6A modification can be used as an important indicator for tumor prognosis. Generally, high levels of m6A modification are associated with poorer prognosis, while low levels of m6A modification predict better survival rates (Qu et al., 2021). By detecting the m6A modification status, it can help predict tumor invasiveness and patient survival rates. Additionally, changes in m6A modification may serve as biomarkers for early tumor diagnosis, thereby improving the effectiveness of early detection and intervention. The expression patterns of methylation-related key genes are associated with the grading of gliomas and have potential uses in prognostic stratification (Chai et al., 2019). Furthermore, the expression of METTL3 is positively correlated with higher glioma grades and poorer prognosis (Chang et al., 2021b).

RNA methylation, represented by m6A modification, is crucial to tumor heterogeneity, treatment response, and prognosis. Further research into its molecular mechanisms can help understand tumor development, optimize treatment strategies, and provide new prognostic evaluation methods for patients. Combining bioinformatics and cancer genomics data, the study of tumor characteristics, RNA modifications, and treatment responses aims to improve prognosis and treatment outcomes.

#### 4.2.2 Cluster two: the role of noncoding RNA in RNA methylation and tumors

This cluster consists of 22 keywords, including expression, gene, epigenetics, mechanism, hypermethylation, noncoding RNA, risk, promoter methylation, association, promoter, disease, inflammation, etc. The central theme of this cluster can be summarized as the role of noncoding RNA in RNA methylation and tumors.

In recent years, the crucial regulatory role of noncoding RNA (ncRNA) in RNA methylation and tumor research has been highlighted. These ncRNAs, especially microRNAs (miRNAs) and long non-coding RNAs (lncRNAs), not only dominate the regulation of gene expression but also significantly influence tumor formation and progression by affecting RNA methylation patterns.

m6A methylation is involved in regulating various aspects of noncoding RNA, such as generation, splicing, transport, degradation, and expression, all demonstrating significant functionality. Simultaneously, abnormal levels of noncoding RNA expression also affect m6A levels. For example, miRNAs can target the METTL3 methyltransferase, affecting m6A modification levels by regulating its binding to mRNA, thereby intervening in stem cell differentiation (Chen et al., 2015). miR-33a affects tumor proliferation processes by targeting METTL3 mRNA (Du et al., 2017). It can directly bind to the 3′UTR region of METTL3 mRNA in tumor cells, leading to decreased mRNA and METTL3 levels (Liu et al., 2018; Dong et al., 2018; Gao et al., 2019). On the other hand, the lncRNA growth arrest-specific 5 (GAS5) acts as a tumor suppressor gene, inhibiting cancer cell proliferation, invasion, migration, epithelial-mesenchymal transition (EMT), and radioresistance. Its antisense RNA, LncRNA GAS5-AS1, enhances GAS5 stability by acting on the demethylase ALKBH5 and regulating GAS5 m6A modification, thereby inhibiting tumor cell proliferation, invasion, migration, and metastasis.

Noncoding RNA as a potential therapeutic target has received significant attention. By specifically acting on conserved sequences of noncoding RNA, reducing m6A-modified transcript levels can affect downstream gene expression, thereby regulating the biological functions of tumor cells.

#### 4.2.3 Cluster three: potential therapeutic targets of RNA methylation in tumors

This cluster consists of 20 keywords, including methylation, gene expression, identification, protein, RNA, activation, transcription, inhibition, chromatin, stem cells, and binding. The central theme of this cluster can be summarized as the potential therapeutic targets of RNA methylation in tumors. As a dynamic RNA modification process, RNA methylation is involved in tumor occurrence and progression and is considered a potential diagnostic marker and therapeutic target for tumors.

The m6A demethylase FTO, as a member of the 2-oxoglutarate (2OG) and iron-dependent dioxygenase (NAOX) family, provides a new pathway for the development of highly specific and efficient inhibitors through substrate specificity and catalytic domain analysis. The first FTO inhibitor, rhein, globally increases cellular mRNA m6A levels by binding to the FTO catalytic domain and blocking the recognition of m6A substrates (Chen et al., 2012). Methoxyacetic acid (MA) and its ester derivative MA2, as highly selective FTO inhibitors, significantly inhibit tumor cell growth and self-renewal and slow tumor growth in mouse models, significantly prolonging survival (Cui et al., 2017). In addition, MO-I-500, as a pharmacological inhibitor of FTO, effectively inhibits cell survival and/or colony formation in TNBC cell lines (Singh et al., 2016). R-2-hydroxyglutarate (R-2HG), a product of mutated isocitrate dehydrogenase 1/2 (IDH1/2), plays an anti-tumor role in leukemia by inhibiting cell proliferation, promoting cell cycle arrest, and apoptosis (Su et al., 2018). Mechanistically, R-2HG directly targets the m6A demethylase FTO and inhibits its catalytic activity, thereby increasing m6A-modified RNA levels in R-2HG-sensitive leukemia cells. Furthermore, the combination of R-2HG with various frontline anti-cancer drugs such as all-trans retinoic acid (ATRA), azacitidine, decitabine, and doxorubicin further enhances leukemia treatment efficacy (Qing et al., 2021). CS1 and CS2 are highly efficient FTO inhibitors screened from 260,000 compounds. They inhibit FTO demethylase activity by selectively binding to and occupying the catalytic pocket of FTO and demonstrate potent anti-tumor effects and high clinical feasibility in various cancers (Su et al., 2020).

In addition to FTO inhibitors, the inhibitor of METTL3, STM2457, as a highly efficient and selective inhibitor, with *in vivo* activity and therapeutic efficacy, marks the first evidence of the effectiveness of RNA methyltransferase inhibitors in cancer therapy. BTYNB, as a novel inhibitor of IGF2BP1, inhibits cell cycle and cancer progression by impairing IGF2BP1-dependent mRNA coding factor stability (Mahapatra et al., 2017; Muller et al., 2020).

In summary, therapeutic strategies targeting m6A methyltransferases show enormous potential as anticancer treatments. Current m6A methylation inhibitors alter the overall level of m6A methylation by targeting enzymes responsible for this process. However, further research is needed to explore whether targeting gene-specific m6A methylation can bring about better therapeutic effects.

#### 4.2.4 Cluster four: the role of RNA methylation in tumor progression and metastasis: a case study of hepatocellular carcinoma and gastric cancer

This cluster comprises 20 keywords, including proliferation, metastasis, progression, growth, hepatocellular carcinoma, invasion, apoptosis, pathway, microRNA, gastric cancer, and migration. The central theme of this cluster can be summarized as the role of RNA methylation in tumor progression and metastasis, using hepatocellular carcinoma and gastric cancer as examples.

Numerous studies have demonstrated the crucial role of m6A modification in the epithelial-mesenchymal transition (EMT) process of hepatocellular carcinoma and gastric cancer (Yue et al., 2019; Zhang et al., 2024). During the EMT process, the m6A modification level of mRNA significantly increases, marking a critical step in cancer cell migration. Downregulation of m6A modification caused by METTL3 deficiency significantly impairs cancer cell migration, invasion, and EMT process. Further m6A sequencing and functional studies have confirmed the regulation of key transcription factor SNAIL by m6A modification during EMT. Additionally, YTH N6-methyladenosine RNA-binding protein 1 mediates m6A-induced snail mRNA translation, further emphasizing the central role of m6A in regulating cancer cell EMT (Lin et al., 2019).

In hepatocellular carcinoma (HCC), METTL3 adds m6A methylation marks to the coding sequence (CDS) and 3′untranslated region (UTR) of key EMT transcription factor Snail, thereby enhancing its translation process. Subsequently, this translation process is further supported by the interaction of YTHDF1 and eukaryotic translation elongation factor 2 (eEF-2), ultimately driving the development of HCC (Lin et al., 2019). Moreover, METTL3-mediated m6A modification stabilizes the RNA transcript of LINC00958, upregulating the expression of hepatoma-derived growth factor (HDGF), thereby promoting HCC progression (Zuo et al., 2020). Bioinformatics analysis based on TCGA database data has revealed that METTL14 may participate in the malignant progression of HCC by regulating m6A-modified genes, including cysteine sulfinic acid decarboxylase (CSAD), glutamate oxaloacetate transaminase 2 (GOT2), and suppressor of cytokine signaling 2 (SOCS2) (Li et al., 2020). The m6A methyltransferase KIAA1429 is significantly upregulated in HCC tissues, promoting the migration and invasion of liver cancer cells by increasing the level of m6A in DNA-binding inhibitor 2 (ID2) mRNA (Cheng et al., 2019). Additionally, the m7G methyltransferase WD repeat domain 4 (WDR4) is abnormally upregulated and promotes tumor metastasis and sorafenib resistance through EMT. Mechanistically, WDR4 enhances the binding of eukaryotic translation initiation factor 2A to CCNB1 mRNA, thereby promoting the translation of cyclin B1 (CCNB1) and exacerbating the progression and metastasis of HCC (Xia et al., 2021).

Based on bioinformatics analysis using the TCGA database, low m6A signal levels are predictive factors for adverse clinical-pathological characteristics of gastric cancer. Decreased m6A RNA methylation levels can activate the oncogenic WNT/PI3K-AKT signaling pathway, thereby promoting the development of GC (Zhang et al., 2019). METTL3 is upregulated in GC, and its high expression is closely associated with poor prognosis. METTL3 mediates m6A modification on the zinc finger MYM-Type domain of mRNA containing ZMYM1 through an HUR-dependent pathway, enhancing its stability. Furthermore, ZMYM1 recruits the CTBP/LSD1/COREST complex to bind to the E-cadherin promoter, promoting the epithelial-mesenchymal transition (EMT) process and cell migration (Yue et al., 2019). Downregulation of METTL3 significantly inhibits proliferation and migration of human GC cells and inactivates the AKT signaling pathway, a finding further validated by Liu et al. (2020). Additionally, low protein expression levels of the demethylase gene FTO in GC patients are closely associated with shortened overall survival, indicating its important role in the progression and metastasis of GC and significant prognostic value (Li Y. et al., 2019).

#### 4.2.5 Cluster five: regulation mechanisms of m6A methylation in leukemia cell differentiation and tumorigenesis

This cluster comprises 14 keywords, including messenger RNA, N6-methyladenosine, m6A, translation, differentiation, RNA methylation, nuclear RNA, METTL3, tumorigenesis, metabolism, demethylase, leukemia, and messenger RNA. The central theme of this cluster can be summarized as the regulation mechanisms of m6A methylation in leukemia cell differentiation and tumorigenesis.

Studies have found that both mRNA and protein expression levels of METTL3 significantly increase in leukemia cells. Loss of METTL3 in human myeloid leukemia cell lines triggers receptor mouse cell differentiation and apoptosis, thereby slowing down the progression of leukemia (Wang et al., 2020). In immune-deficient mouse models, downregulation of METTL3 expression leads to cell cycle arrest, blockade of leukemia cell differentiation, and inhibition of leukemia formation. Both knockout and overexpression of METTL3 result in upregulation of WTAP protein expression levels. METTL14 shows high expression levels in normal hematopoietic stem/progenitor cells (HSPCs) and acute myeloid leukemia (AML) cells carrying t (11q23), t (Yang et al., 2015; Li Q. et al., 2017), or t (Jiang et al., 2020; Peng et al., 2022) translocations (Weng et al., 2018). During bone marrow differentiation, the expression level of METTL14 gradually decreases. Silencing of METTL14 promotes myeloid terminal differentiation of normal HSPCs and AML cells while inhibiting the survival and proliferation of AML cells (Bansal et al., 2014). Additionally, FTO has been found to promote leukemia oncogene-mediated cell transformation and leukemia development, while inhibiting all-trans retinoic acid-induced differentiation of AML cells and regulating the expression of its target genes, such as ankyrin repeat and SOCS box-containing 2 (ASB2) and retinoic acid receptor alpha (RARA), through lowering m6A levels in mRNA transcripts (Li Z. et al., 2017). Furthermore, R-2-hydroxyglutarate (R-2HG) exhibits inhibitory effects on leukemia cells by inhibiting their proliferation/survival, promoting cell cycle arrest, and apoptosis.

### 4.3 Limitations

This study employed a bibliometric analysis method aimed at comprehensively dissecting and visualizing the knowledge structure and dynamic evolution of RNA methylation-related research in the cancer field. However, several limitations were encountered during this process. Firstly, our study primarily relied on the widely recognized and authoritative WoSCC comprehensive database for literature screening. While this ensured the credibility and reliability of the data, it may have overlooked potentially important literature not included in this database. Additionally, our study was limited to English-language literature, which might restrict our comprehensive understanding of cancer RNA methylation research in different linguistic backgrounds globally, potentially overlooking important research findings in other language environments.

## 5 Conclusion and future perspectives

This study provides a comprehensive analysis of current trends, global collaboration patterns, research hotspots, and emerging frontiers in RNA methylation-related research in cancer for the first time. From 2014 to 2023, the literature volume on RNA methylation-related research in cancer has shown a steady overall growth trend. Over the past 5 years, related research has entered a period of rapid development. Co-occurrence cluster analysis of keywords reveals five research directions as the current focus: the role of RNA methylation in the heterogeneity, treatment response, and prognosis of tumors; the role of non-coding RNA in RNA methylation and tumors; potential therapeutic targets of RNA methylation in tumors; the role of RNA methylation in tumor proliferation, invasion, and metastasis; and the regulatory mechanisms of m6A methylation in leukemia cell differentiation and tumorigenesis. The rapid development of m6A modification research signifies significant progress in molecular biology. Epitranscriptomics is an important subfield of epigenetics that can help reveal the biological mechanisms underlying cancer development and may provide new targets for cancer therapy. In summary, we believe that the shifting hotspots in cancer RNA methylation research primarily stem from rapid advancements in molecular biology technologies and urgent clinical needs. With the development of high-throughput sequencing and other cutting-edge techniques, scientists can now more precisely decipher the role of RNA methylation in cancer, especially its pivotal function in tumor heterogeneity, treatment response, and prognosis. Additionally, novel discoveries regarding the regulation of RNA methylation by non-coding RNAs, coupled with the rapid rise of m6A methylation research, have introduced fresh hotspots to the field. These changes not only shed light on the molecular mechanisms underlying cancer development but also offer promising new targets for cancer therapy. Bibliometric analysis provides a comprehensive understanding of RNA methylation-related research in cancer and offers valuable references for future researchers in this field.

## Data Availability

The original contributions presented in the study are included in the article/supplementary material, further inquiries can be directed to the corresponding author.
